# Acute respiratory failure in renal transplant recipients: a single ICU experience

**DOI:** 10.1186/2197-425X-3-S1-A904

**Published:** 2015-10-01

**Authors:** A Ulas, Ş Kaplan, P Zeyneloglu, A Torgay, A Pirat, M Haberal

**Affiliations:** Baskent University, Ankara, Turkey

## Introduction

Pulmonary complications after renal transplantation have been reported to range from 3% to 17%. Renal transplant recipients requiring ICU admission for acute respiratory failure (ARF) are associated with high mortality and graft loss rates.

## Objectives

To evaluate renal transplant recipients admitted to ICU in order to identify incidences and causes of ARF in the postoperative period and compare clinical features and outcomes between those with and without ARF.

## Methods

We retrospectively screened 540 consecutive adult recipients who received their grafts in a single transplant center from 01/2005-03/2015. Among them, patients admitted to ICU during this period were included for the analysis of those with ARF defined as severe dyspnea, respiratory distress, decreased SpO_2_ (< 92%), hypoxemia (PaO_2_< 60mmHg) or hypercapnia (PaCO_2_>60mmHg) on room air or requirement of noninvasive or invasive mechanical ventilation. Demographic, clinical and laboratory data were collected. APACHE II and SOFA scores at ICU admission and lengths of ICU, hospital stay and mortality were assessed.

## Results

Among the 540 adult renal transplant recipients, 55 (10.7%) were admitted to ICU, including 26 (47.3%) admitted for ARF. Mean APACHE II and SOFA scores of those with ARF on admission were 19.7 ± 11.8 and 5.4 ± 3.7, respectively and mean patient age was 42.4 ± 12.6 years with 81% males. Median time from transplantation to ICU admission was 10 months (0-67). The leading causes of ARF were bacterial pneumonia (56%) and cardiogenic pulmonary edema (44%). Acinetobacter baumannii was isolated in 15% of the patients. Mean partial pressure of arterial oxygen to fractional inspired oxygen ratio was 174 ± 59, invasive mechanical ventilation was used in 13 patients (50%) and noninvasive mechanical ventilation was used in 8 patients (31%). Shock at ICU admission was seen in 11 patients (42.3%) and vasopressors were needed in half of them. A history of acute rejection before ICU admission was seen in 10 patients (38.5%). RRT was administered in 13 patients (50%). RRT was more frequently used in patients with ARF when compared to those without ARF (50% vs 23%, p = 0.04). The overall mortality was 16.4%. Patients admitted for ARF had similar lengths of ICU, hospital stays and mortality when compared to those without ARF (p > 0.05 for all). Regarding patients with ARF, mortality was significantly high in those with shock at ICU admission (45.5% vs 6.7%, p = 0.02), with higher SOFA scores on days 1,2,3 (p = 0.001, p < 0.001, p < 0.001) and with diagnosis of pneumonia (38% vs 0%, p = 0.02).

## Conclusions

ARF accounted almost half of renal transplant recipients admitted to ICU in the postoperative period and main causes were bacterial pneumonia and cardiogenic pulmonary edema. Mortality of patients admitted for ARF was similar to those without ARF, but survival was worse in patients with shock at ICU admission, higher SOFA scores and diagnosis of pneumonia.

